# Silent myocarditis in systemic sclerosis detected by cardiovascular magnetic resonance using Lake Louise criteria

**DOI:** 10.1186/s12872-017-0619-x

**Published:** 2017-07-17

**Authors:** Sophie Mavrogeni, Loukia Koutsogeorgopoulou, Georgia Karabela, Efthymios Stavropoulos, Gikas Katsifis, John Raftakis, Sotiris Plastiras, Michel Noutsias, George Markousis-Mavrogenis, Genovefa Kolovou

**Affiliations:** 1 Onassis Cardiac Surgery Center, 50 Esperou Street, 175-61 P.Faliro, Athens, Greece; 2 Pathophysiology Department, Laikon Hospital, Athens, Greece; 3 Athens Naval Hospital, Athens, Greece; 4 Asklipion Voulas Hospital, Athens, Greece; 5 Department of Internal Medicine III, Division of Cardiology, Angiology and Intensive Medical Care, University Hospital Halle, Martin-Luther-University Halle, Ernst-Grube-Straße 40, D-06120 Halle (Saale), Germany

**Keywords:** Cardiovascular magnetic resonance, Systemic sclerosis, Myocarditis, Myocardial fibrosis

## Abstract

**Background:**

Systemic sclerosis (SSc) is an autoimmune disease characterized by microvascular abnormalities, inflammation and fibrosis. We hypothesized that myocarditis may be diagnosed in asymptomatic SSc, undergoing routine cardio-vascular magnetic resonance (CMR) for fibrosis assessment, using the Lake Louise criteria: T2 ratio, early (EGE) and late gadolinium enhanced (LGE) images.

**Methods:**

Eighty-two asymptomatic SSc, diagnosed according to American College of Rheumatology criteria, aged 43 ± 5 yrs., 62 with diffuse (dSSc) and 20 with localized (lSSc) systemic sclerosis were evaluated by CMR, performed at 1.5 T scanner, according to Lake Louise criteria.

**Results:**

CMR documented normal biventricular function in all SSc. However, 7/62 (11.2%) with dSSc and 2/20 (10%) with lSSc, had CMR signs of myocarditis according to Lake Louise criteria, without any clinical cardiac symptom. In these 9 patients, T2 ratio, EGE ratio and LGE (positive in all 9 SSc) were 2.8 ± 0.5%, 8 ± 3% and 5 ± 3% of LV mass, respectively. No correlation between CMR and blood inflammatory indices (C-reactive protein and erythrocyte sedimentation rate), cardiac troponin T, disease characteristics or type of SSc was identified. A repeat CMR at 6 months, after treatment with prednisone and azathioprine, showed normalisation of the acute inflammation CMR indices.

**Conclusions:**

Silent myocarditis may be diagnosed using the Lake Louise paper criteria in SSc patients without cardiac symptoms, has no correlation with blood inflammatory indices, cardiac troponin or disease characteristics. CMR is a promising tool to diagnose silent myocarditis in SSc and monitor the response to immunosuppressive treatment.

## Background

Systemic sclerosis (SSc) is an autoimmune disease characterized by microvascular abnormalities, inflammation and increased fibrosis of all organs, including the heart [1]. Asymptomatic cardiac disease can be presented in up to 80% of SSc patients [2–6] and is included between the three major causes of death in SSc [4]. However, clinically overt heart disease in SSc is less frequent, and was documented only in 20–25% of them [3].

Myocarditis, fibrosis, pulmonary hypertension and blood vessel abnormalities are considered as the main etiologic factors of cardiac involvement in SSc. Myocarditis is a contributor of recent-onset cardiac disease in SSc, and if timely treated, the progression to further cardiac damage can be avoided by immunosuppression [7]. However, the true incidence of myocarditis in asymptomatic SSc has never been assessed.

Cardiovascular Magnetic Resonance (CMR), a non-invasive, non-radiating, operator independent technique, has been successfully used for the evaluation of myocardial fibrosis in SSc [8, 9]. The ability to perform tissue characterisation makes CMR an excellent diagnostic tool to detect myocarditis either due to infective [10] or connective tissue diseases, including SSc [11–15]. The International Consensus Group on CMR Diagnosis of Myocarditis achieved consensus among CMR experts and published the Lake Louise criteria about recommendations on the current state-of-the-art use of CMR for myocarditis. The recommendations include indications for CMR in patients with suspected myocarditis, CMR protocol standards, terminology for reporting CMR findings, and diagnostic criteria for myocarditis [10]. According to Lake Louise criteria, oedema evaluation was based on the ratio of myocardial against skeletal muscle signal intensity in STIRT2 images. Early (EGE) was based on ECG-triggered T1-W multislice spin-echo images obtained in axial orientation with identical parameters before and after an intravenous bolus of 0.1 mmol/kg Gd-DTPA within 1 min of injection in the same area as in STIRT2 images. Finally, late gadolinium-enhanced images (LGE) were assessed from images taken 15 min later after another injection of 0.1 mmol/kg Gd-DTPA using an inversion recovery sequence. The examination was considered as positive for myocarditis if 2/3 examined indices are positive [10]. Given that the expected tissue pathology in active myocarditis includes intracellular and interstitial oedema, capillary leakage, hyperaemia and – in more severe cases - myocytolysis and replacement fibrosis, the Lake Louise criteria provide a reliable diagnostic approach to diagnose myocarditis.

We hypothesized that the Lake Louise criteria for myocarditis [10] may be also positive in asymptomatic SSc patients. The aim of our study was to diagnose the potential presence of CMR indices positive for myocarditis according to Lake Louise criteria in asymptomatic SSc.

## Methods

### Patients

Eighty-two consecutive SSc without cardiac symptoms, diagnosed according to 2013 American College of Rheumatology criteria [16] (ACR score 13 ± 2), aged 43 ± 5 yrs., 62 with diffuse (dSSc) and 20 with localized (lSSc) systemic sclerosis were evaluated. Disease characteristic were presented in Table 1. Exclusion criteria included contraindications to CMR, heart/renal/liver impairment, severe dyspnoea due to lung involvement, pulmonary hypertension, pregnancy and hypersensitivity to gadolinium. All patients gave a written informed consent and the protocol was approved by the Onassis Cardiac Surgery Center ethics committee.

**Table 1:** Disease characteristics of SSc population at the time of CMR evaluation

Disease characteristics	dSSc (*n* = 62)	lSSc (*n* = 20)
Female/Male	55/7	17/3
Age (years)	43 ± 2	38 ± 4
Disease duration (months)	5 ± 3	6 ± 1
Current smokers	5	1
Hypertension	8	1
Diabetes	3	0
Hyperlipidemia	6	0
Lung involvement	50	0
Raynaud phenomenon	58	1
Digital ulcers	45	0
Pulmonary hypertension	0	0
GI involvement	28	0
Arthritis	5	0
ESR, mm/h (normal 3–18)	10 ± 2	13 ± 5
CRP, mg/L (normal 2–8)	4 ± 2	2 ± 3
Cardiac troponin T (cTnT), ng/mL (normal 0.01 ± 0.03)	0.006 ± 0.007	0.008 ± 0.007
mRSS	20 ± 6	-
Anti-centromere antibodies, n (%)	0	12
Anti-topoisomerase 1 antibodies, n (%)	53	0
Calcium channel blockers	57	8
Methotrexate	15	0
Prednizolone	0	0
Azathioprine	3	0
Cloroquine	2	1
NSAID	18	3

*ESR* Erythrocyte sedimentation rate

*CRP* C-reactive protein

*Mrss* Modified Rodnan skin score

*NSAID* Non-steroidal anti-inflammatory drug

### Methods

The presence of myocarditis and the left ventricular systolic function were evaluated by CMR. Myocarditis was documented using STIR T2-weighted (T2-W), T1-weighted before and early after contrast media injection (EGE) and late enhanced images (LGE). The study was considered as positive for myocarditis, according to Lake Louise criteria [10].

### A. CMR evaluation of inflammation

Cardiovascular magnetic resonance examination was performed in a 1.5 T Philips Intera system using STIR T2-weighted (T2-W), T1-weighted (T1-W) before and early after contrast media injection (EGE) and late enhanced images (LGE). ECG-triggered, STIR T2-W multislice spin-echo sequence was performed in axial orientation and the signal ratio was calculated from the left ventricular myocardium and the skeletal muscle in the same slice. ECG-triggered T1-W multislice spin-echo images were also obtained in axial orientation with identical parameters before and after an intravenous bolus of 0.1 mmol/kg Gd-DTPA. Measurements were performed after 1 min of injection of Gd-DTPA for early gadolinium enhancement (EGE) in the same area as in T2-W. Immediately after this, 0.1 mmol/kg Gd-DTPA was given again and late gadolinium-enhanced images (LGE) were taken 15 min later, using an a 3D–T1-TFE sequence, preconditioned with a 180 degrees inversion pulse (flip angle =15°, TE = 1.4 msec, TR = 5.5 ms, TI 225 to 275 ms as individually optimized to null myocardial signal, matrix 256X192 and slice thickness = 5 mm). Images were analyzed according to previously described protocols [10].

### B. CMR functional study

For each subject, localizing scans were obtained to define the long (2-chamber) axis of the left ventricle. A mid ventricular short axis view was prescribed, and used to plan a 4-chamber view. The short axis orientation was then defined accurately, perpendicular to both the 2- and 4-chamber views. To cover the entire left ventricle, 10 contiguous (gap = 0 mm) short axis slices were acquired in each study. The imaging sequence was a 2D, multi-phase (16 cardiac phases were acquired per cardiac cycle resulting to a temporal resolution of 47 ms for a heart rate of 80 beats/min), steady-state free-precession (SSFP) sequence (TE = 1.5 ms, TR = 3.1 ms, flip angle = 70°, slice thickness = 8 mm, acquired in-plane spatial resolution = 1.8 mm × 2.0 mm) characterized by the application of balanced gradients in all directions [10].

### C. Image analysis

In T2-W the signal ratio was measured from the region of interest covering the left ventricular myocardium as well as within a skeletal muscle in the same slice.

xIn T1-W early enhancement (EGE), which reflects hyperaemia and capillary leak as a marker of inflammation, the early myocardial enhancement was measured from the region of interest covering the left ventricular myocardium as well as within a skeletal muscle in the same slice [10].

To assess the contrast-enhanced images (LGE), all short-axis slices from base to apex were evaluated for areas of normal (completely nulled) myocardium [9, 10]. Mean signal intensity and standard deviation (SD) was derived and a threshold of >6 SD exceeding the mean was used to define areas of LGE. Summing the planimetered areas of LGE in all short- axis slices yielded the total volume, which was expressed as a proportion of total LV myocardium (% LGE). The LGE analysis was performed by one experienced reader and reviewed and by a second expert reader, with both of the readers blinded to patient’s identity and clinical history. Any discrepancies between the 2 readers was then adjudicated by a senior reader with >10 yrs. of CMR experience, also blinded to patient’s identity and clinical history [9].

Cine images were used for the evaluation of left and right ventricular ejection fraction (LVEF/RVEF), using the MRI-MASS (Medis, Leiden, the Netherlands) software was used and the readers were blinded to the clinical data [11].

### Statistical analysis

All measurements were expressed as mean ± SD. Statistical significance of the differences was investigated using unpaired Student’s T-test. Correlation between variables was sought with Pearson’s correlation coefficient. For non-parametric data, the Mann-Whitney test and Spearman’s correlation coefficient were used respectively. Statistical significance was considered for *p* < 0.05.

## Results

**Fig. 1:**
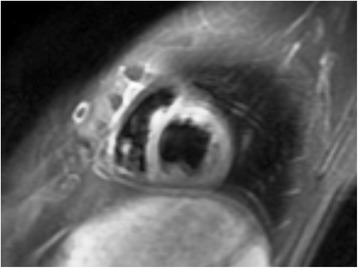
Short axis STIR T2 showing evidence of oedema in an SSc patient without clinical cardiac symptoms

**Fig. 2:**
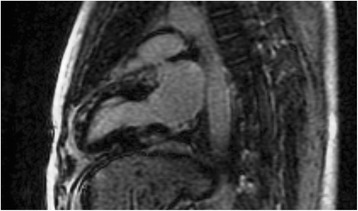
Two-chamber inversion recovery image showing fibrotic lesions in the anterior and inferior wall of LV, in a patient with SSc, compatible with asymptomatic myocarditis

In our study, CMR documented normal biventricular function in all SSc evaluated. However, 7/62 with dSSc and 2/20 with lSSc, although they had no cardiac symptoms/signs, were positive for myocarditis, according to Lake Louise criteria [10]. In all 9 SSc patients, 3/3 indices were positive for myocarditis. T2 ratio (Fig. 1), EGE and LGE (Fig. 2) were 2.8 ± 0.5, 8 ± 3 and 5 ± 3% of LV mass, respectively. No patient with 2/3 positive indices was identified. In SSc patients without evidence of myocarditis T2 ratio was 1.6 ± 0.2 and 1.5 ± 0.3, EGE 1.16 ± 0.9 and 0.6 ± 0.4, LGE 1.6 ± 2.8 and 0.7 ± 1.4 for dSSc and lSSc, respectively.

There was no correlation between the CMR findings and the blood inflammatory indices (C-reactive protein/CRP and erythrocyte sedimentation rate/ESR), the cardiac troponin T (cTnT) measured at the time of CMR evaluation, the disease characteristics or type of SSc was identified. CMR findings of SSc were presented in Table 2.

**Table 2:** CMR findings of the SSc patients studied

Characteristics	dSSc (*n* = 62)	lSSc (*n* = 20)
LVEDV (ml)	117.9 ± 27.9	119.9 ± 19.2
LVESV (ml)	42.6 ± 19.8	40.9 ± 9.5
LVEF (%)	64.7 ± 9.4	63.5 ± 5.7
RVEDV (ml)	110.8 ± 30.9	108.5 ± 21.7
RVESV (ml)	54.4 ± 25.9	46.1 ± 11.8
RVEF (%)	55 ± 11	57 ± 8.5
LGE (%LV)	2 ± 2.9	1 ± 1.77
T2 ratio	1.8 ± 0.4	1.6 ± 0.4
EGE	1.9 ± 2.2	1.4 ± 2.6

*LVEDV* Left ventricular enddiastolic volume

*LVESV* Left ventricular endsystolic volume

*LVEF* Left ventricular ejection fraction

*RVEDV* Right ventricular enddiastolic volume

*RVESV* Right ventricular endsystolic volume

*RVEF* Right ventricular ejection fraction

*EGE* Early Gd enhancement

*LGE* Late Gd enhancement

Treatment with prednisone 20 mg poqd and azathioprine 100 mg poqd started after the positive CMR result for myocarditis was available. A follow up CMR was performed in all SSc patients after 6 months of immunosuppression. These follow up CMR investigations showed complete normalisation of CMR indices indicative of acute myocarditis (T2 ratio = 1.2 ± 2, EGE = 2.5 ± 0.8 and LGE = 3 ± 2% of LV mass).

## Discussion

In our study, using the Lake Louise criteria, we documented that 10% of patients with either dSSc or lSSc presented CMR evidence of myocarditis, although they had no cardiac symptoms/signs and positive inflammatory or cardiac biomarkers. No correlation between type of SSc, disease duration, inflammatory indices or cTnT levels and CMR findings was identified. After 6 months of immunosuppression a complete normalization of CMR indices indicative of acute inflammation was identified.

Although myocardial involvement in SSc was usually attributable to non-inflammatory causes, there are early reports presenting SSc patients with evidence of myocarditis and good response to the combination of either corticosteroids and azathioprine [11] or corticosteroids and cyclophosphamide [12]. Our results are in agreement with the majority of previous studies detecting myocarditis in SSc [7–9, 13–15, 17, 18]. However, we identified a significant lower incidence of myocarditis in comparison with other studies [15]. This discrepancy is probably due to the lack of T1, T2 mapping and Extracellular Volume (ECV) assessment. Indeed, native T1 mapping is superior to T2-weighted imaging in detecting myocardial oedema [19]. T1 mapping and ECV can also detect diffuse fibrosis and were well correlated with histology [20]. However, in SSc, it is difficult to distinguish if an increase in T1 mapping is due to myocarditis or to diffuse myocardial fibrosis, which is the “trademark” of the disease, as both can increase native T1 mapping values and ECV. The application of T1 mapping as an oedema index in SSc can potentially lead to overestimation of myocarditis’ incidence and “overmedicalisation” of these patients, since it is known that myocarditis in SSc demands prompt treatment with corticosteroids and immunosuppressive drugs [7]. In this context, the application of T2 mapping seems more rational for the detection of recent myocardial inflammation in SSc. This concept is further supported by studies documenting that only T2 mapping seems to be superior, when compared with standard CMR parameters, global myocardial T1, and ECV for assessment of active myocarditis in patients with recent-onset heart failure and reduced LVEF [21]. The application of new CMR indices, mainly of T2 mapping that is the best indicator of myocardial oedema, may further increase the incidence of asymptomatic myocarditis and serve as a prognostic indicator in asymptomatic SSc patients [22].

Although parametric imaging was not available in these SSc patients, recent unpublished data from our group after assessment of 24 SSc patients evaluated by both Lake Louise criteria and parametric imaging in our department showed an increase of T2 mapping in 15/24 SSc with values ranging between 62 and 75 msec, STIR T2 ratio > 2 in 17/24 and native T1 values ranging between 1110 and 1235 mesc in all 22/24 SSc patients. These very preliminary data seem rather support of the use of T2 instead of T1 mapping as an index of recent inflammation in SSc.

Another important issue is the lack of correlation between the blood inflammatory indices and cTnT and CMR indices. However, this is not unusual because lack of such correlation was also identified in both infective [23] and autoimmune myocarditis [8, 13, 24]. This lack of correlation further emphasizes the role of CMR as a powerful diagnostic tool that can provide direct tissue characterization beyond clinical and laboratory findings and potentially modify both rheumatic and cardiac treatment.

Endomyocardial biopsy (EMB), although it carries serious limitations due to its invasive nature, interobserver variability and sampling error, is considered as the diagnostic “gold standard” in clinical scenarios suggestive of myocarditis [25, 26]. Autoimmune myocarditis is likely to be present in our SSc patients, but an overlapping of potential presence of viral myocarditis can not be excluded in the absence of EMB. However, in the clinical setting of our SSc patients, the EMB indication did not fulfil class I or II evidence level [27]. Therefore, we lack a comparative analysis between CMR and EMB in our cases. However, the improvement of CMR indices after immuno-suppressive treatment was strongly supportive of autoimmune myocarditis.

The published literature in the field supports the crucial role of CMR in the diagnosis of myocarditis, as a contributor of heart disease in SSc [7–9, 13–18]. However, there is one study supporting that although CMR is considered as the gold standard in the diagnosis of myocarditis, its role was controversial or falsely negative in half of patients with biopsy-proven myocarditis [28]. Keeping in mind that fibrosis is the hallmark of myocardial disease in SSc, and its pathogenesis is multifactorial, due to microvascular ischemia, spasm and inflammation, this controversy seems rather expectable. Indeed, in SSc, fibrosis may not be used as an independent criterion of myocarditis, since it represents the result of both myocarditis and fibrotic disease independent of inflammatory processes, typical of SSc. On the other hand, the diagnosis of asymptomatic myocarditis in SSc is extremely significant, because it demands prompt rheumatic treatment to avoid future irreversible cardiac lesions [7]; furthermore, although it is easy to diagnose the presence of clinically overt myocarditis, which usually presents with concurrent myositis in SSc [7], it remains rather difficult to achieve this target in asymptomatic cases [8, 13, 18]. The presence of 3 positive CMR indices in all our patients emphasizes the clinical role of Lake Louise criteria in the early detection of asymptomatic myocarditis in SSc. However, due to the pathophysiology of fibrosis in SSc, these criteria may need modifications with regard to myocarditis’ diagnosis in SSc. In these patients, maybe all 3 indices should be used for a positive diagnosis and not only the 2/3, as it is the current recommendation of the Lake Louise criteria.

### Limitations of the study

The following limitations may be discussed for this study:

1. The patients’ sample was small
2. T1 and T2 mapping was not performed, because these sequences were not available at the time of the CMR examination at our center. However, we should mention that although these indices represent an important part of current CMR research, they are not widely available and standardization of the relevant protocols and normal values are still under evaluation [29].
3. Endomyocardial biopsy (EMB) for comparison with CMR findings was not performed, because our patients were not eligible for EMB, due to lack of symptoms and normal biventricular function [30]. Furthermore, the sensitivity of EMB is hampered by e.g. a substantial sampling error due among others to the patchy distribution of this disease (sampling error) [31], and the shortcomings of the currently applied methodology of some laboratories [32].

## Conclusions

Silent myocarditis may be diagnosed using the Lake Louise criteria in a considerable percentage of SSc patients presenting without clinical cardiac symptoms, and has no correlation with blood inflammatory indices (CRP, ESR) or further clinical disease characteristics. CMR signs of myocarditis frequently normalize 6 months under after immunosuppressive treatment with corticosteroids and immunosuppressive treatment.

## Abbreviations

CMR: Cardiovascular Magnetic Resonance
CRP: C reactive protein
EMB: Endomyocardial biopsy
ESR: Erythrocyte sedimendation rate
JACC: Journal of American Society of Cardiology
LVEF: Left ventricular ejection fraction
SSc: Systemic sclerosis
SSFP: Steady-state free-precession sequence
T1-W: T1- weighted
T2-W: T2- weighted
TE: Echo time
TR: Repetition time
